# A Narrative Review of da Vinci Single-Port Versus Multi-Port Robotic Surgery in Elderly or Frail Urological Cancer Patients

**DOI:** 10.3390/cancers18152448

**Published:** 2026-07-30

**Authors:** Maria Chiara Sighinolfi, Vincenzo Cavarra, Giuseppe Pallotta, Francesco Rossi, Nicoletta Testori, Simone Assumma, Enrico Panio, Filippo Turri, Carlo Gandi, Giuseppe Palermo, Mauro Ragonese, Pierluigi Russo, Nazario Foschi, Filippo Gavi, Giuseppe Colloca, Luca Tagliaferri, Chiara Ciccarese, Roberto Iacovelli, Angelo Totaro, Marco Racioppi, Bernardo Rocco

**Affiliations:** Fondazione Policlinico Gemelli IRCCS, Largo Agostino Gemelli 8, 00168 Rome, Italygiuseppeferdinando.colloca@policlinicogemelli.it (G.C.);

**Keywords:** robotic surgery, prostate cancer, renal cancer, elderly, frail patients, single-port, geriatric assessment

## Abstract

Background: Elderly and frail patients undergoing surgery for urological cancer have increased vulnerability to postoperative complications, delayed recovery, and functional decline. The da Vinci Single-Port (SP) platform may reduce surgical stress by enabling regionalized extraperitoneal, retroperitoneal, or transvesical access through a single incision. Objective: The objective was to provide a narrative review of the comparative evidence on da Vinci SP versus conventional multi-port (MP) robotic surgery in elderly or frail urological cancer patients. Methods: A structured search was performed to identify comparative studies reporting outcomes in patients defined as elderly by chronological age (≥65 years) or as frail by a validated frailty index. Findings are reported separately according to the vulnerability construct that was actually measured. Because of procedural and methodological heterogeneity, a narrative synthesis was performed, and the risk of bias was appraised qualitatively along the domains of the ROBINS-I tool. Results: Only three retrospective comparative studies are currently available: two on robot-assisted radical prostatectomy (one in patients aged ≥65 years and one in patients stratified by the 5-item modified frailty index) and one on robot-assisted partial nephrectomy in patients aged ≥65 years. Across these studies, SP surgery was associated with fewer early postoperative complications, shorter length of stay, and more favourable recovery-related endpoints, and in the frailty-stratified study the apparent protective effect increased with frailty burden. All estimates are those published by the original authors, are reported descriptively, and were not pooled. Importantly, in none of the three studies was the SP platform compared with MP surgery performed through the same access route: SP procedures were predominantly extraperitoneal or retroperitoneal and MP procedures were predominantly transperitoneal, so the effect of the platform cannot be separated from that of the access route. Conclusions: The available data are compatible with a reduction in early perioperative morbidity after SP robotic surgery in selected elderly or frail urological cancer patients, particularly when the platform enables extraperitoneal or retroperitoneal access. Because this evidence rests on three retrospective studies at serious risk of bias, the findings are preliminary and hypothesis-generating rather than confirmatory. Prospective studies incorporating geriatric screening, validated frailty metrics, patient-reported recovery, cost-effectiveness, and long-term oncological outcomes are needed.

## Simple Summary

Prostate and kidney cancers are most often diagnosed in older adults, and many of these patients are frail, meaning that they recover from surgery less easily than younger or fitter people. Surgeons now have access to the single-port robot, a newer system that works through one small incision rather than several and that can often reach the prostate or the kidney without entering the abdominal cavity and without steeply tilting the patient head-down during the operation. In this narrative review we bring together the only three studies published so far that have directly compared this single-incision approach with the standard multi-incision one in older or frail patients with urological cancer. These reports describe fewer early complications, shorter hospital stays and quicker recovery, with the largest difference in the frailest patients. All three studies, however, look back at patients already treated, and in most of them the single-port operation was also performed through a different route of access, so the observed benefits may reflect both the single-port robot and the change in access route rather than use of the robot alone; the findings should be read as an early signal to be confirmed rather than as proof. We also outline how this surgical option can be combined with a careful assessment of each older patient before surgery.

## 1. Introduction

The progressive ageing of the population is reshaping surgical decision-making in urological oncology. Prostate cancer and renal cell carcinoma are predominantly diseases affecting older adults, and a growing proportion of patients are now diagnosed at an age at which potentially curative treatment remains appropriate but physiological reserve is heterogeneous. Chronological age alone is an insufficient criterion for excluding surgery. Older patients of the same age can range from robust and independent to vulnerable, frail, cognitively impaired, malnourished, or burdened by competing comorbidities. Frailty is an age-independent syndrome characterized by reduced resilience to stressors and is associated with higher postoperative complications after common urological procedures [1,2,3]. In this setting, the value of surgical innovation should not be judged only by the ability to reproduce oncological outcomes, but also by its capacity to reduce the biological cost of treatment.

The da Vinci SP platform represents one of the most relevant recent developments in minimally invasive urological surgery. Its rationale is rooted in the long-standing attempt to reduce morbidity by performing complex procedures through fewer access sites. Earlier laparoendoscopic single-site approaches showed that single-incision surgery could improve cosmesis and potentially reduce abdominal wall trauma, but their diffusion was limited by instrument crowding, loss of triangulation, poor ergonomics, and a steep technical learning curve [4,5]. The purpose-built SP system was designed to overcome these limitations: a single robotic arm carries a flexible high-definition camera and three double-articulating instruments through a 2.5-cm multichannel cannula, and the patient cart is conceived to work in narrow spaces [6,7,8,9,10,11]. Dedicated solutions such as the so-called bubble-chamber configuration and the floating docking technique were introduced to optimize the internal working space, particularly in shallow extraperitoneal or retroperitoneal fields [12]. SP surgery should therefore not be considered a merely cosmetic modification of multi-port robotic surgery, but a platform that changes access strategy and workspace geometry; several of its proposed benefits derive from access and docking rather than from the number of incisions alone.

In radical prostatectomy, the SP platform can be used through transperitoneal, Retzius-sparing, extraperitoneal, transperineal, and transvesical routes [13,14,15,16]. The transperitoneal approach closely resembles standard multi-port robot-assisted radical prostatectomy (MP-RARP), with Trendelenburg positioning and a supra-umbilical access, and still requires peritoneal entry, which may be problematic in elderly or frail patients with cardiopulmonary limitations or previous abdominal surgery [15,16]. Regionalized approaches are where SP technology may have its greatest clinical meaning. Extraperitoneal SP-RARP can often be performed in the supine position with minimal or no Trendelenburg through a low midline incision, limiting bowel manipulation and peritoneal violation; in cohorts not selected for age, it has been associated with earlier recovery, shorter hospitalization, lower analgesic requirements, and comparable early oncological outcomes when compared with transperitoneal MP-RARP [17,18,19,20,21,22,23]. Transvesical SP-RARP takes this concept further, approaching the prostate through a contained pelvic route that avoids the peritoneal cavity and may reduce the need for mechanical ventilation or steep positioning in selected cases, at the price of a technically demanding dissection [24,25], whereas Retzius-sparing SP-RARP has been associated with favourable early continence in selected series [25,26,27]. SP prostate surgery is therefore best regarded as a family of approaches rather than as a single operation, with the optimal route depending on cancer characteristics, prostate volume, need for lymphadenectomy, previous surgery, patient anatomy, and surgeon expertise.

In kidney surgery, SP partial nephrectomy can be performed transperitoneally or retroperitoneally, and contemporary practice shows growing interest in retroperitoneal and anterior retroperitoneal access, which provides direct exposure of the kidney without bowel mobilization [28,29,30,31,32,33,34]. Comparative series and meta-analyses suggest perioperative outcomes broadly similar to multi-port surgery, with possible advantages in length of stay, pain, and recovery in selected patients [35,36,37,38,39,40]. Other uro-oncological applications, including radical nephrectomy, nephroureterectomy, cystectomy, and selected reconstructive procedures, have also been described, and multi-quadrant access without repositioning or re-docking is feasible [41,42,43]. Across all these indications, however, most available studies are retrospective and derive from single-centre or high-volume experiences, and long-term oncological and functional data remain limited.

A balanced appraisal must also consider the drawbacks of the SP platform and the enduring strengths of multi-port robotics. The SP system provides a smaller instrument set, lower overall traction force, and a single assistant channel, so bedside assistance, specimen extraction, stapling, and the control of intraoperative bleeding may be more demanding than with multi-port robotics [11]. Conversely, the multi-port platform remains the reference option for large or anatomically complex tumours, extended lymphadenectomy, and demanding reconstructive steps; it offers a wider range of instruments; and it is far more widely available. Adoption of the SP platform also requires a dedicated learning curve that differs from that of standard robotic procedures, and its per-procedure cost is higher. These considerations are directly relevant to the interpretation of the present review, because the reported advantages of SP surgery have so far been generated almost exclusively in high-volume institutions with mature robotic programmes and established enhanced-recovery pathways.

For elderly and frail patients, these platform characteristics generate a clinically coherent hypothesis. If a single-port approach reduces peritoneal violation, incision burden, Trendelenburg exposure, pain, opioid requirement, delayed mobilization, and length of hospitalization, its relative benefit should be greatest in patients with limited physiological reserve, in whom an early complication can trigger a cascade of delirium, immobility, deconditioning, readmission, loss of independence, and inability to receive subsequent oncological therapies. The purpose of the present narrative review is to appraise the available comparative evidence on da Vinci SP versus conventional multi-port robotic surgery in elderly or frail urological cancer patients, to distinguish what has been demonstrated in patients selected by chronological age from what has been demonstrated in patients selected by a validated frailty instrument, and to make explicit the methodological limitations that currently constrain any comparative claim.

## 2. Materials and Methods

**Search Strategy and Information Sources**

**Figure 1:**
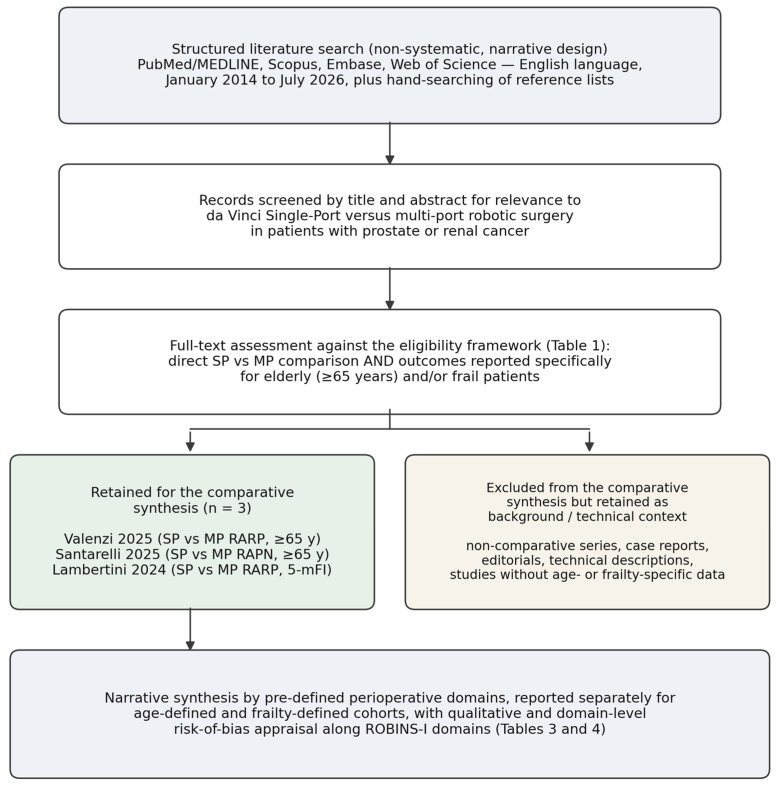
Flow of the literature selection process for the present narrative review. The diagram summarizes the criteria and the logic by which studies were retained for the comparative synthesis or used as background material.

To ensure a comprehensive overview of the literature, a structured and iterative search was performed across PubMed/MEDLINE, Scopus, Embase, and Web of Science, covering publications from January 2014, when the current SP platform entered clinical use, to the last update performed during manuscript revision (May 2026). The search strategy used free-text combinations of the following terms: da Vinci Single Port, single port, multi-port, multiport, robotic, urology, radical prostatectomy, partial nephrectomy, renal cell carcinoma, prostate cancer, elderly, older, frail, frailty, 5-item modified frailty index, postoperative complications, readmission, same-day discharge, and length of stay. The search was supplemented by cross-referencing the bibliographies of retrieved articles and relevant reviews. Only articles published in English were considered. Consistent with the narrative design of this work, the review was not registered in PROSPERO, PRISMA-based record accounting was not applied, and no formal counts of identified, screened, and excluded records are reported; the logic of the selection process is summarized in Figure 1.

**Study Selection and Eligibility Framework**

Given the narrative design of this review, article selection was guided by author consensus based on clinical relevance to the core question, rather than by a rigid screening algorithm. Strict conceptual boundaries were nevertheless maintained: priority was given to comparative studies directly evaluating da Vinci SP robotic surgery against multi-port robotic approaches in elderly and/or frail cohorts. To safeguard the validity of comparative statements, non-comparative series, case reports, and studies lacking age- or frailty-specific data were excluded from the comparative synthesis, although they were retained where necessary to provide technical context or clinical reasoning (Table 1; Figure 1). Two authors independently identified the eligible comparative studies, and any disagreement was resolved by discussion with a senior author.

**Data Synthesis and Methodological Appraisal**

To capture the clinical nuances relevant to vulnerable patient populations, the narrative is structured around pre-defined perioperative domains: 30- and 90-day postoperative complications, major complications, readmissions, length of stay, same-day discharge, operative time, estimated blood loss, access route, and perioperative safety.

Due to the substantial heterogeneity across the available literature—specifically regarding surgical procedures, definitions of patient vulnerability, patient-matching strategies, and outcome reporting—quantitative meta-pooling was neither feasible nor methodologically sound. Findings are therefore presented via structured descriptive synthesis based on original study estimates.

To ensure a transparent critical appraisal despite the narrative format, the methodological limitations and potential biases of the included studies were evaluated qualitatively. This appraisal was framed conceptually around the domains of the ROBINS-I tool for non-randomized comparative studies (confounding, selection of participants, classification of interventions, deviations from intended interventions, missing data, measurement of outcomes, and selection of the reported result). It is offered as an interpretative lens to contextualize the certainty of the reviewed evidence rather than as a formal systematic-review assessment; the resulting narrative and domain-level judgements are reported in Table 2, Table 3 and Table 4.

**Table 1:** PICO framework and eligibility criteria.

PICO Element	Definition
Population	Patients undergoing robotic surgery for prostate or renal cancer and identified as vulnerable by either of two distinct constructs: chronological age (≥65 years) or frailty measured with a validated instrument (5-item modified frailty index). The two constructs are related but not interchangeable and are reported separately throughout the review.
Intervention	da Vinci Single Port robotic surgery.
Comparator	Conventional multi-port robotic surgery. In all available studies the multi-port comparator was predominantly transperitoneal, whereas single-port procedures were predominantly extraperitoneal or retroperitoneal, so platform and access route are confounded.
Outcomes	30-day postoperative complications, major complications, 90-day complications, readmission, length of stay, same-day discharge, operative time, estimated blood loss, and perioperative safety.
Synthesis	Narrative synthesis, reported separately according to the vulnerability construct actually measured; no meta-analysis because of the small number of studies and the heterogeneity of procedure, access route, and definition of vulnerability. Qualitative and domain-level risk-of-bias appraisal along ROBINS-I domains (Table 3 and Table 4).

**Table 2:** Comparative studies of da Vinci Single Port versus multi-port robotic surgery in elderly or frail urological cancer patients. n = number of patients.

Study	Setting	Population	Comparison	Main Findings Favoring SP	Follow-Up; Adjustment Method
Valenzi et al., 2025 [44]	Radical prostatectomy	Elderly patients ≥ 65 yr	MP-RARP n = 54 vs. SP-RARP n = 82	30-day complications: 35.2% vs. 15.9%; 90-day complications: 38.9% vs. 17.1%; LOS: 35 vs. 18 h; same-day discharge: 16.7% vs. 54.9%; SP protective at 30 and 90 days.	30- and 90-day perioperative follow-up; multivariable logistic regression for complications (age-defined subgroup analysis).
Lambertini et al., 2024 [45]	Radical prostatectomy	Frail patients stratified by 5-mFI	Matched MP-RARP n = 126 vs. SP-RARP n = 126	In 5-mFI ≥ 2: overall complications 52% vs. 23%; major complications 19.6% vs. 8.2%; SP protective for overall and major complications; benefit increased with frailty burden.	30-day perioperative follow-up; 1:1 propensity-score matching, with frailty-stratified and multivariable analyses.
Santarelli et al., 2025 [46]	Partial nephrectomy	Elderly patients ≥ 65 yr	MP-RAPN n = 42 vs. SP-RAPN n = 44	Operative time: 206 vs. 173.5 min; EBL: 100 vs. 50 mL; LOS: 2 vs. 0 d; SP independently protective for 30-day complications.	30-day perioperative follow-up; multivariable logistic regression for complications.

**Table 3:** Qualitative risk-of-bias assessment of included comparative studies.

Study	Design	Key Strengths	Main Concerns	Overall Risk of Bias
Valenzi et al., 2025 [44]	Retrospective comparative cohort	Elderly-specific analysis; multivariable regression; clinically relevant endpoints	Non-randomized; possible selection and learning-curve bias; discharge protocols may influence LOS and same-day discharge	Serious
Lambertini et al., 2024 [45]	Retrospective propensity-score matched cohort	Frailty measured with 5-mFI; matched comparison; frailty-stratified results	Residual confounding; extraperitoneal SP compared with transperitoneal MP; single-center high-volume experience	Serious
Santarelli et al., 2025 [46]	Retrospective comparative cohort	Elderly-specific RAPN analysis; multivariable regression; renal cancer focus	Non-randomized; tumor complexity and access selection may confound results; short follow-up	Serious

**Table 4:** Domain-level appraisal of the included comparative studies according to the domains of the ROBINS-I tool.

ROBINS-I Domain	Valenzi et al., 2025 [44]	Santarelli et al., 2025 [46]	Lambertini et al., 2024 [45]
Confounding	Serious	Serious	Serious
Selection of participants	Moderate	Moderate	Moderate
Classification of interventions	Low	Low	Low
Deviations from intended interventions	Moderate	Moderate	Moderate
Missing data	Moderate	Moderate	Moderate
Measurement of outcomes	Moderate	Moderate	Moderate
Selection of the reported result	Moderate	Moderate	Moderate
Overall judgement	Serious	Serious	Serious

## 3. Results

Three comparative studies met our eligibility criteria [44,45,46]. Two evaluated robot-assisted radical prostatectomy for prostate cancer and one evaluated robot-assisted partial nephrectomy for renal cell carcinoma. Together, they represent the entirety of the direct comparative evidence on SP versus multi-port robotic surgery in elderly or frail urological cancer patients; no randomized trial was identified. Two of the three studies defined the vulnerable population by chronological age (≥65 years) [44,46] and one by a validated frailty instrument, the 5-item modified frailty index (5-mFI) [45]; their findings are therefore presented separately below. All figures are those published by the original authors and are reported descriptively, without pooling; where confidence intervals or exact *p* values were not available in the source publication, proportions and medians are reported without inferential qualifiers.

### 3.1. Evidence in Cohorts Defined by Chronological Age

Valenzi et al. compared SP-RARP and MP-RARP in elderly patients with localized prostate cancer [44]. In the subgroup aged 65 years or older, SP-RARP was associated with a shorter hospital stay and a higher rate of same-day discharge: length of stay was 18 h after SP-RARP versus 35 h after MP-RARP, and same-day discharge was achieved in 54.9% versus 16.7% of elderly patients. The difference in postoperative complications was also clinically meaningful, with 30-day complications in 15.9% of SP-RARP patients versus 35.2% of MP-RARP patients and 90-day complications in 17.1% versus 38.9%. On multivariable analysis, the SP approach remained independently associated with a lower risk of complications at both 30 and 90 days; effect sizes with confidence intervals were not reported for all endpoints in the source publication. The authors also described one conversion to open surgery in the multi-port group because of haemodynamic instability during Trendelenburg positioning, a detail that underlines the importance of positioning tolerance in elderly patients.

Santarelli et al. evaluated SP versus multi-port robot-assisted partial nephrectomy in elderly patients with cN0M0 renal cell carcinoma [46]. Among patients aged 65 years or older, the SP cohort had a higher rate of extraperitoneal (retroperitoneal) access, a shorter operative time (median 173.5 versus 206 min), lower estimated blood loss (50 versus 100 mL), and a shorter length of stay (median 0 versus 2 days). The crude 30-day complication rate was numerically lower in the SP group (7% versus 19%), and multivariable analysis identified the SP approach as independently protective for 30-day postoperative complications. These findings suggest that the recovery advantages described for SP surgery are not confined to pelvic surgery and may also apply to nephron-sparing renal cancer surgery when a retroperitoneal access is feasible, although the difference in access route between the two cohorts is itself part of the comparison.

### 3.2. Evidence in the Cohort Defined by a Validated Frailty Index

The study by Lambertini et al. is the only study that measured frailty with a validated instrument rather than using age as a proxy [45]. In a propensity-score matched cohort undergoing radical prostatectomy, 126 patients treated with extraperitoneal SP-RARP were compared with 126 patients treated with transperitoneal MP-RARP. Frailty was quantified with the 5-mFI, which includes chronic obstructive pulmonary disease or pneumonia, congestive heart failure, dependent functional status, hypertension requiring medication, and diabetes. The most relevant finding was an interaction between frailty burden and surgical approach: among patients with 5-mFI ≥ 2, overall complications were lower after SP-RARP than after MP-RARP (23% versus 52%), as were major complications (8.2% versus 19.6%). In the most vulnerable strata the difference was more pronounced, with 30-day complications of 25% versus 58% among patients with 5-mFI 3 and 31% versus 63% among patients with 5-mFI ≥ 4. Extraperitoneal SP-RARP was an independent protective factor for both overall and major complications. This study is of particular interest because it is compatible with the hypothesis that the benefit of reduced invasiveness increases as physiological reserve decreases; being a single retrospective matched analysis; however, it requires independent confirmation, and its findings should not be extrapolated to cohorts defined only by chronological age.

### 3.3. Platform Effect Versus Access-Route Effect

One methodological caveat applies to the whole of this evidence base and is deliberately foregrounded here, rather than confined to the Discussion, because it conditions the interpretation of every estimate reported above. In none of the three studies was the SP platform compared with multi-port surgery performed through the same access route. In both prostatectomy studies the SP procedures were predominantly or exclusively extraperitoneal, while the multi-port comparator was transperitoneal [44,45], and in the partial nephrectomy study the SP cohort included a substantially higher proportion of retroperitoneal access than the multi-port cohort [46]. What is being compared is therefore a combination of platform, number of incisions, access route, patient positioning, anaesthetic requirements, and institutional discharge pathways. The isolated contribution of the SP platform cannot be quantified from the available data, and the differences described in Section 3.1 and Section 3.2 should be read as differences between two surgical strategies rather than between two robotic systems.

### 3.4. Overall Pattern Across Studies

Considered together, and within the limits described above, the three studies point in the same direction: a reduction in early postoperative morbidity in the SP groups, observed despite differences in procedure, population, and endpoint definitions. In prostate cancer surgery, the reported differences concern shorter hospitalization, higher feasibility of outpatient care, and fewer early complications in elderly or frail patients; in renal cancer surgery, they concern shorter operative time, lower blood loss, shorter hospitalization, and a lower adjusted risk of 30-day complications. No signal of compromised early oncological safety was reported, although long-term cancer-control data are immature and were not an endpoint of any of the three studies. The consistency of direction across studies should be interpreted with caution, since the three cohorts share the same structural limitations and, as discussed below, originate from a small number of centres.

## 4. Discussion

The discussion of SP robotic surgery in elderly and frail urological cancer patients should begin with patient selection. Contemporary prostate cancer guidance emphasizes that individual life expectancy, health status, frailty, and comorbidity should guide treatment decisions rather than chronological age alone [47]. The EAU-EANM-ESTRO-ESUR-ISUP-SIOG guidelines recommend formal health-status screening in men older than 70 years using the Geriatric 8 (G8), Mini-COG, and Clinical Frailty Scale (CFS), and they recommend comprehensive geriatric assessment for patients with a G8 score ≤ 14 [47,48,49,50,51,52]. This framework is directly applicable to surgical decision-making. Fit older patients and vulnerable patients whose impairments can be reversed should not be denied standard curative treatment solely because of age. Conversely, frail patients with irreversible impairment may require adapted treatment, watchful waiting, or symptom-directed care depending on cancer aggressiveness and life expectancy [47].

A practical preoperative pathway for elderly urological cancer patients should therefore include at least three levels. First, life expectancy should be estimated using age, comorbidity, cancer risk, and functional measures. Gait speed is a simple marker of survival in older adults and can complement oncological risk stratification [53]. Second, frailty screening should be formalized. The G8 captures nutrition, weight loss, mobility, neuropsychological problems, body mass index, polypharmacy, self-rated health, and age; a score ≤ 14 should trigger geriatric referral or comprehensive geriatric assessment [48,54]. The Mini-COG can identify cognitive impairment, which is relevant to informed consent, postoperative delirium risk, and adherence to recovery instructions [55]. The CFS provides a common language for grading frailty from very fit to terminally ill and has been associated with postoperative mortality and discharge destination [51,52]. Third, geriatric domains should be mapped to modifiable interventions: nutritional supplementation for weight loss or low body mass index, prehabilitation for sarcopenia or slow gait speed, medication review for polypharmacy, optimization of cardiopulmonary disease, delirium-prevention strategies, social support planning, and shared decision-making that includes the patient and, when appropriate, caregivers [56,57,58,59].

Within this framework, SP surgery should be considered a technical option that may reduce perioperative stress in selected patients, not a substitute for geriatric assessment. The studies included in this review suggest that the SP approach may shift the risk-benefit balance in favor of surgery for some elderly or frail patients by reducing early complications and length of stay [44,45,46]. However, this does not mean that every older patient should undergo SP surgery. Patients with aggressive cancer and preserved function may benefit from standard curative treatment, whether SP or multi-port, whereas patients with severe irreversible frailty may not benefit from any major operation. The most clinically meaningful target population may be the intermediate group: patients with localized or locally advanced urological cancer who have enough life expectancy to benefit from treatment but enough vulnerability that reducing surgical stress is likely to matter.

Several mechanisms may explain the observed reduction in postoperative morbidity. First, extraperitoneal and retroperitoneal SP approaches avoid or minimize peritoneal violation and bowel manipulation. This may reduce ileus, nausea, delayed diet, lymphocele-related morbidity, and systemic effects of transperitoneal pneumoperitoneum. Second, extraperitoneal SP-RARP can be performed in supine position with minimal or no Trendelenburg, whereas transperitoneal MP-RARP typically requires steep Trendelenburg and lithotomy. Older patients with heart failure, chronic obstructive pulmonary disease, obesity, glaucoma, cerebrovascular disease, or limited cardiopulmonary reserve may tolerate a regionalized supine approach better. Third, a single incision and smaller abdominal wall footprint may reduce pain, opioid requirement, and delayed mobilization. In elderly patients, opioid-sparing recovery is not merely a comfort outcome; it may reduce constipation, nausea, falls, urinary retention, and delirium. Fourth, shorter hospitalization and same-day discharge may reduce exposure to nosocomial complications, sleep disruption, immobility, and hospital-acquired delirium, provided that discharge pathways are safe and social support is adequate. It should be stressed that these mechanisms, although biologically plausible, remain hypothetical in this population: none of the three included studies measured postoperative delirium, cognitive outcomes, opioid consumption, functional trajectories, or loss of independence, and no conclusion about the prevention of functional decline in elderly or frail patients can be drawn from the available data.

The frailty-specific data are of particular interest. In the Lambertini study, the difference between SP and MP surgery was modest in fitter patients and widened as the 5-mFI increased [45]. This gradient is biologically plausible: a fit patient may recover well after either robotic approach, whereas a patient with limited reserve may be pushed beyond that reserve by additional pain, positioning stress, ileus, or prolonged hospitalization. In this sense, a lower-stress operation might be regarded as a procedure-level form of prehabilitation, which cannot reverse frailty but may reduce the magnitude of the stressor. This remains a hypothesis generated by a single retrospective matched cohort, in which the SP arm also differed by access route, and it has not been replicated by independent groups. Future studies should test the concept prospectively, incorporating baseline frailty instruments, postoperative functional trajectories, patient-reported recovery, delirium, discharge destination, and loss of independence, rather than limiting outcomes to Clavien-Dindo complications.

The renal cancer data also support a patient-centered interpretation. Elderly patients with small renal masses often face a choice between active surveillance, thermal ablation, partial nephrectomy, and radical nephrectomy. The decision depends on tumor complexity, baseline renal function, competing mortality risk, and patient preferences. In frail or comorbid patients, active surveillance or ablation may be appropriate, but partial nephrectomy remains important when oncological control and nephron preservation are priorities. SP-RAPN may be especially attractive when a retroperitoneal or anterior retroperitoneal route provides direct tumor access while avoiding bowel mobilization [28,29,30,31,32,33,34,46]. Nevertheless, renal hilar control, ischemia time, tumor complexity, and surgeon experience remain central. The SP approach should not be selected if it compromises resection quality, renorrhaphy, hemostasis, or the ability to manage intraoperative complications.

The current evidence also has important limitations. All included studies were retrospective and non-randomized. Even when propensity-score matching was used, residual confounding is likely. Surgeons may preferentially select healthier patients, smaller tumors, less complex anatomy, or more favorable body habitus for SP surgery during the learning phase. Conversely, high-volume centers may use SP specifically for complex patients with hostile abdomen or frailty, which could bias results in the opposite direction. Learning curve effects are difficult to separate from platform effects because SP adoption often occurs in expert robotic centers with standardized recovery pathways. Differences in access route are another major issue: many comparisons are not pure SP-versus-MP comparisons but extraperitoneal SP versus transperitoneal MP comparisons. Therefore, the apparent benefit may reflect a combination of platform, incision burden, access route, positioning, anesthesia, and institutional discharge protocols.

A further limitation concerns the concentration of the available evidence within a small number of research environments. The three included studies share investigators, and several of the background publications on SP outcomes originate from the same collaborative networks, including the Single Port Advanced Research Consortium (SPARC) [29,30,34,35,44,45,46]. This is an expected consequence of the limited diffusion of the platform, since only a few centres have accumulated the case volume required for comparative analyses, and it does not in itself invalidate the findings. It does, however, reduce the independence of the evidence: methodological choices, definitions, indications, and reporting practices are likely to be correlated across studies, so the consistency of results is less reassuring than the same consistency observed across unrelated groups, and the possibility that outcomes reflect the surgical culture of a few high-volume centres cannot be excluded. The authors of the present review were not involved in any of the included studies. Confirmation from independent institutions, ideally with different enhanced-recovery pathways and case mix, is a priority.

Learning-curve effects are equally difficult to disentangle from platform effects. The learning curve of SP surgery differs from that of standard multi-port robotic procedures and varies across operations and access routes, as recently reviewed for both multi- and single-port urological surgery [60]. Because the available comparative series were generated during a phase of active adoption, their outcomes may reflect not only the platform but also case selection during the learning phase, team familiarity, and the maturity of institutional recovery protocols. This consideration also limits the transferability of the reported results to centres beginning their SP experience, in which a benefit of comparable magnitude should not be assumed a priori.

Outcome reporting is also heterogeneous. The included studies focused on early complications, length of stay, and discharge, but little data is available on long-term functional recovery, continence, potency, renal function, oncological endpoints, readmissions beyond 30 or 90 days, patient-reported pain, opioid consumption, delirium, or return to baseline activities. Same-day discharge is a valuable endpoint, but it can be influenced by institutional culture, insurance systems, home distance, caregiver availability, and patient preference. For elderly patients, safe discharge should be interpreted together with readmission, emergency visits, falls, urinary retention, catheter problems, and caregiver burden. Future research should standardize these endpoints and should include geriatric outcomes such as postoperative delirium, change in ADL/IADL, discharge to rehabilitation, and independence at 30 and 90 days.

Cost-effectiveness is a further dimension that the present evidence leaves entirely unaddressed. The SP platform requires a dedicated system, single-use multichannel access devices, and a specific instrument set, so the per-procedure cost is generally higher than that of multi-port robotic surgery, and none of the included studies reported costs, resource use, or economic endpoints. It is plausible that shorter hospitalization, higher rates of same-day discharge, and fewer early complications partly or entirely offset the higher procedural cost, particularly in older patients, in whom complications and prolonged admissions are expensive; it is equally plausible that they do not, especially outside high-volume centres or in health systems where outpatient pathways are less developed. Formal economic evaluations, incorporating readmissions, emergency visits, and post-discharge care, are needed before any efficiency argument can be made for or against the platform in this population.

A meta-analysis was not performed in this review because the available evidence is too sparse and heterogeneous. Pooling one partial nephrectomy study with two radical prostatectomy studies would generate a summary effect that is statistically fragile and clinically difficult to interpret. Even within prostatectomy, one study focused on age and another on 5-mFI-defined frailty, and the comparator was often transperitoneal multi-port surgery rather than an alternative extraperitoneal multi-port approach. A narrative synthesis with risk-of-bias appraisal is therefore more transparent. As additional studies emerge, a meta-analysis may become appropriate if outcomes, frailty definitions, and access routes are sufficiently comparable.

From a clinical perspective, the evidence supports a selective rather than indiscriminate adoption of SP surgery. The ideal candidate is an older or frail patient with a surgically treatable urological cancer, meaningful life expectancy, and a specific reason why reduced access trauma, avoidance of the peritoneal cavity, reduced Trendelenburg, or outpatient recovery would improve the perioperative risk profile. For prostate cancer, this may include patients with localized disease, preserved life expectancy, cardiopulmonary vulnerability, prior abdominal surgery, or high priority for early mobilization. For renal cancer, this may include patients with small renal masses appropriate for partial nephrectomy in whom retroperitoneal access is technically favorable. For all patients, the operation should be performed by teams with adequate SP experience, established conversion strategies, and integrated geriatric and anesthetic pathways.

This review has implications for trial design. Future prospective studies should stratify patients by age, G8, CFS, Mini-COG, CCI, ADL/IADL, and nutritional status. Randomized trials may be difficult because platform availability and surgeon expertise vary, but prospective multicenter registries with standardized geriatric variables could substantially improve evidence quality. Comparisons should separate platform effects from access-route effects by including transperitoneal SP, extraperitoneal SP, transperitoneal MP, and extraperitoneal MP cohorts when feasible. Outcomes should include oncological safety, major complications, failure-to-rescue, delirium, functional decline, opioid use, same-day discharge failure, readmission, patient-reported recovery, and cost-effectiveness. Such data would allow clinicians to determine not only whether SP surgery is safe, but which patient is most likely to benefit. Looking further ahead, platform comparisons should be placed within the broader technological evolution of minimally invasive and image-guided therapy, which now includes intraoperative fluorescence-guided lesion navigation and magnetically actuated micro-robotic delivery systems [61,62]; in parallel, artificial intelligence-based predictive models are being developed to refine risk stratification and outcome prediction in oncology [63] and could, in principle, help to identify prospectively the older or frail patients most likely to benefit from a reduced-stress surgical strategy.

Overall, the present evidence suggests that da Vinci SP surgery may reduce early perioperative morbidity in elderly or frail urological cancer patients, particularly when the platform enables an extraperitoneal or retroperitoneal route that avoids physiologically stressful elements of conventional transperitoneal surgery. The evidence is promising but not definitive. The central message is not that SP surgery should replace multi-port robotic surgery, but that it may expand the ability to tailor minimally invasive cancer surgery to the physiological profile of older adults. Framed in the terms proposed by the reviewers of this manuscript, the appropriate status of these observations is preliminary and hypothesis-generating: they justify prospective evaluation, and they do not establish the comparative effectiveness of one robotic platform over the other.

## 5. Risk of Bias and Certainty of Evidence

The appraisal identified an overall serious risk of bias for the available evidence, primarily because all included studies are retrospective and non-randomized (Table 3 and Table 4). Confounding by indication is the most important concern: patient anatomy, tumour complexity, surgeon preference, time period, access route, and institutional enhanced-recovery pathways may all have influenced the assignment to SP or MP surgery. Propensity-score matching in the frailty prostatectomy study mitigated but did not eliminate this limitation, and it could not balance the difference in access route [45]. Selection bias is also possible, because SP cohorts frequently reflect early adoption in high-volume centres. Classification of the intervention was generally reliable, since platform and access route were clearly described. Measurement of outcomes was acceptable for objective endpoints such as complications, length of stay, and estimated blood loss, although complication ascertainment in retrospective datasets may be incomplete. Selective reporting could not be excluded, since study protocols were not available. The small number of eligible reports and the differences in procedures precluded any meaningful assessment of publication bias.

Judgements refer to the comparison of single-port versus multi-port robotic surgery for early postoperative complications and were reached by consensus of the review authors. They are presented as a structured interpretative aid within a narrative review and not as a formal systematic-review, risk-of-bias assessment. Ratings follow the ROBINS-I terminology (low, moderate, serious, and critical risk of bias).

On balance, the certainty of the evidence for comparative effectiveness should be considered low to very low. The consistency of direction across the three studies is nevertheless compatible with a real effect, supports clinical equipoise, and justifies prospective evaluation; it should not be read as confirmation, both because the studies share the same structural limitations and because they originate from a small number of collaborating centres.

## 6. Conclusions

Da Vinci SP robotic surgery is associated with favourable early perioperative outcomes compared with multi-port robotic surgery in elderly or frail patients undergoing radical prostatectomy or partial nephrectomy. In the study that stratified patients by a validated frailty index, the apparent advantage was largest in those with the highest frailty burden; because this gradient derives from one retrospective study, it should be read as hypothesis-generating rather than established. The evidence base as a whole rests on three retrospective studies at serious risk of bias, in which the SP platform was almost always coupled with an extraperitoneal or retroperitoneal access route; the evidence is therefore preliminary and hypothesis-generating rather than confirmatory, and it does not establish the superiority of one platform over the other.

Placed in the context of the wider literature, these findings are consistent with the recognized advantages of access strategies that avoid the peritoneal cavity and steep Trendelenburg positioning, and they extend to older and frail patients a rationale so far supported mainly in unselected cohorts. Their practical implication is that the surgical approach may be one of the modifiable components of perioperative stress, to be weighed within a structured geriatric evaluation rather than in isolation, and not that any older patient should be directed towards a specific platform. For research, priority should be given to prospective and ideally multicentre studies that separate platform from access route; adopt validated frailty instruments instead of chronological age; involve centres independent of the groups that generated the current evidence; and measure geriatric, patient-reported, and economic outcomes alongside oncological endpoints.

## Data Availability

No new data were created or analyzed in this study. Data sharing is not applicable to this article.

## Abbreviations

The following abbreviations are used in this manuscript:

5-mFI	5-item modified frailty index
ADL/IADL	activities of daily living/instrumental activities of daily living
CCI	Charlson Comorbidity Index
CFS	Clinical Frailty Scale
COPD	chronic obstructive pulmonary disease
EBL	estimated blood loss
G8	Geriatric 8 screening tool
LESS	laparoendoscopic single-site surgery
LOS	length of stay
MP	multi-port
MP-RAPN	multi-port robot-assisted partial nephrectomy
MP-RARP	multi-port robot-assisted radical prostatectomy
PICO	Population, Intervention, Comparator, Outcome
RAPN	robot-assisted partial nephrectomy
RARP	robot-assisted radical prostatectomy
RCC	renal cell carcinoma
ROBINS-I	Risk Of Bias In Non-randomised Studies of Interventions
SP	single-port (da Vinci SP platform)
SPARC	Single Port Advanced Research Consortium
SP-RAPN	single-port robot-assisted partial nephrectomy
SP-RARP	single-port robot-assisted radical prostatectomy
